# Review of Cutting Temperature Measurement Methods

**DOI:** 10.3390/ma16196365

**Published:** 2023-09-22

**Authors:** Piotr Cichosz, Paweł Karolczak, Kamil Waszczuk

**Affiliations:** Faculty of Mechanical Engineering, Wroclaw University of Science and Technology, St. Łukasiewicza 5, 50-371 Wrocław, Poland; piotr.cichosz@pwr.edu.pl (P.C.); pawel.karolczak@pwr.edu.pl (P.K.)

**Keywords:** cutting temperature, temperature measurements, cutting control, measurement disturbances

## Abstract

During the cutting process, large quantities of emitted heat are concentrated on a small surface area of the interface between the workpiece and the cutting edge. The resultant very high temperature significantly affects the tool life. Knowledge of maximum temperatures to be expected on the cutting edges is important, as it allows the cutting conditions to be adjusted in such a manner that the critical value of thermal resistance is not exceeded for the cutting material. In effect, the maximum effectiveness of the working process is maintained. This article offers a systematic presentation of methods used in cutting temperature measurements. It discusses their advantages and disadvantages, as well as the usefulness of the individual methods in different types of machining processes. It also points to the possibility of methodological errors which significantly reduce measurement accuracy. The above issues are believed to justify a discussion of different cutting temperature measurement methods. The conclusions here presented may be of particular importance to researchers interested in the field, especially in high-efficiency machining, new cutting materials and cutting-edge protective coatings, as well as various methods for cutting fluid applications. They may allow a more informed selection of measurement methods most suitable for particular situations.

## 1. Introduction

**Figure 1:**
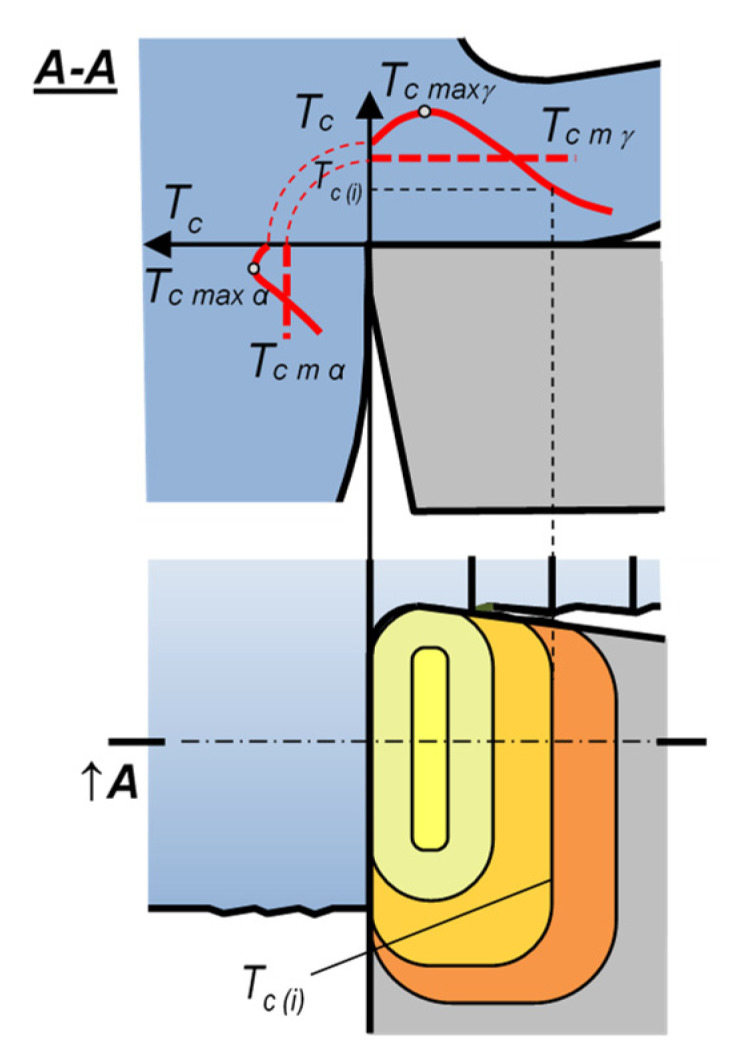
Temperature distribution on the tool edge.

**Cutting temperature *T_c_*** may be defined and interpreted differently. Typically, it is defined as the temperature of the cutting-edge surface [1]. It can also apply to mean values on particular surfaces or to maximum values in particular locations. Figure 1 shows a tool edge with a representatively labeled schematic temperature distribution. The labels in the figure are: *T_c max_*—maximum temperature on the cutting-edge surfaces, *T_c max_*_(*α*,*γ*)_—maximum temperature on the flank face (*α*) or the rake face (*γ*), respectively, *T_c m_*_(*α*,*γ*)_—mean temperature on the edge–workpiece interface, including and excluding the flank surface. In the majority of publications, the term *cutting temperature* by default indicates the maximum value on the surfaces of the cutting edge, unless specified otherwise [2,3,4]. Some other publications employ the term *cutting temperature* to denote the mean value across the entire edge–workpiece interface [5]. Measurements of the maximum temperature on the cutting edge require point measurement methods and are thus substantially more difficult than measurements of mean temperatures [6].

The identification of cutting temperatures is difficult for a number of reasons, in-cluding:

- The volume of the cutting zone is typically limited to several tens of mm^3^.
- Temperatures in the cutting zone show very high gradients (2D and 3D), with values on the order of ∆*T_c_* = 2000 °C/mm [7]; these become particularly steep in the case of tool edges manufactured of ceramic materials having very low thermal conductivity [8].
- Significant differences between thermal conductivity coefficients λ [W/m K] are observed for tool materials (ceramics—λ = ≈15.5, diamond—λ = ≈140) and for the workpiece materials (titanium—λ = ≈22, aluminum—λ = ≈200) [9].
- Values of friction coefficients, thermoelectric properties, and emission coefficients for individual layers of anti-wear coatings forming thermal barriers are different from each other and from the tool material, causing potential disturbances in measurements, in particular, when the layers become discontinued due to wear; the influence of such disturbances on the phenomena related to cutting temperatures is difficult to estimate [10].
- Chip breakers and control grooves on the rake face of the tool edge affect the generation and distribution of heat in the cutting zone, potentially leading to incorrect comparative interpretations of cutting temperatures between different tool edges [11].
- The conditions of heat generation and distribution are affected by different methods employed in the cooling of the cutting zone, including the type of coolant, the direction and intensity of its application, or the use of minimal-quality lubrication system (MQL) [12].
- Highly dynamic phenomena occur in the cutting zone—for example, for a cutting speed of 300 m/min, the workpiece particle passes a 10 mm long cutting zone in approximately 2 ms.
- The temperature can only be identified for a certain area rather than for a specific point due to substantial surface areas of “measurement points” in the case of pyrometers and of pixels in the case of thermal images [13].
- The emission and reflection coefficients of the surface depend on a great number of factors [14].
- Processing fluids and chips, as well as the movement of heat-emitting surfaces, cause disturbances in the transfer of thermal radiation.
- The positioning of sensors on the tool edge and signal acquisition are problematic, in particular when the cutting operation is performed with tools or workpieces rotating at high speeds [15].
- Openings or slots accommodating the temperature sensors or “windows” for infrared radiation transfer may disturb the heat distribution, and thus the temperature distribution, in the cutting zone.
- Electric faults caused by chips occur during measurements in the majority of methods which employ natural thermocouples comprising the tool or the workpiece [16].
- Dielectricity of the tool or workpiece material limits the use of some methods based on natural thermocouples.
- Access to the cutting zone is difficult, and the cutting zone often moves dynamically within the processing space of the cutting machine, e.g., in the case of drilling, slot milling, or high-speed milling [17].
- The tool edge is subject to wear, which affects the amount of heat generated during cutting operations and increases the edge–workpiece interface, thus hindering the identification of mean or maximum cutting temperatures; this factor is of particular importance in measurements based on natural or semi-artificial thermocouples [18].
- Wear of the tool edge is increased in locations where the thermocouples protrude from the cutting surfaces of the tool, leading to local temperature changes.
- The distance between the measurement point and the cutting surface changes due to tool wear [19].
- Potential reflections from heat and light sources present in the vicinity of the cutting machine occur on the surfaces in the case of temperature measurements with emissivity-based methods.
- Boundary conditions are uncertain in calculation methods used in the identification of temperature distributions [20].

The aim of temperature measurements is frequently not to find the accurate value, but rather to identify the influence of a particular factor, e.g., the hardness of the workpiece material, on the temperature changes. In such cases, some test conditions can be disregarded, e.g., the occasionally problematic identification of the emissivity characteristics for the investigated surface in high temperatures and at progressive oxidation, etc. [21,22]. Errors resulting from emissivity inaccurately assumed for a particular surface may become largely canceled in both measurements, and thus their influence on the temperature difference will be insignificant.

The above difficulties in the identification of cutting temperatures may occur with various intensity depending on the measurement method, the test conditions, and the cutting method. Without consideration paid to such disturbances, temperature measurements may cause false or misinterpreted results. Research publications seldom provide information about whether the tests were performed in conditions allowing the results to be considered reliable or even about which area of the cutting zone is described by the temperature measurement results [23,24,25].

## 2. Methods of Cutting Temperature Measurement

A number of methods allow the calculation or experimental identification of cutting temperatures. They can be classified by various criteria, for example [26]:

By the **phenomenon** describing the thermal condition of the object:

- Thermal balance (calorimetric method);
- Change of chip color during cutting or after cooling;
- Changing color of a special paint (chemical thermocolor method);
- Application to the tool edges of thin layers of different materials having known melting points;
- Changes of metallographic structure;
- The Seebeck, Peltier, Thomson (thermocouple) effect;
- Change of electric resistance (resistance thermometers, thermistors);
- Infrared emission (pyrometric, thermal vision methods).
- By the **zone**, which served for the identification, e.g.,:
- Point;
- Line;
- Surface;
- Volume.

By **extrapolation methods** and **calculation methods**:

- From mathematical models estimated experimentally;
- From physical models, e.g., with the use of finite elements method (FEM) or finite difference method (FDM).

**Figure 2:**
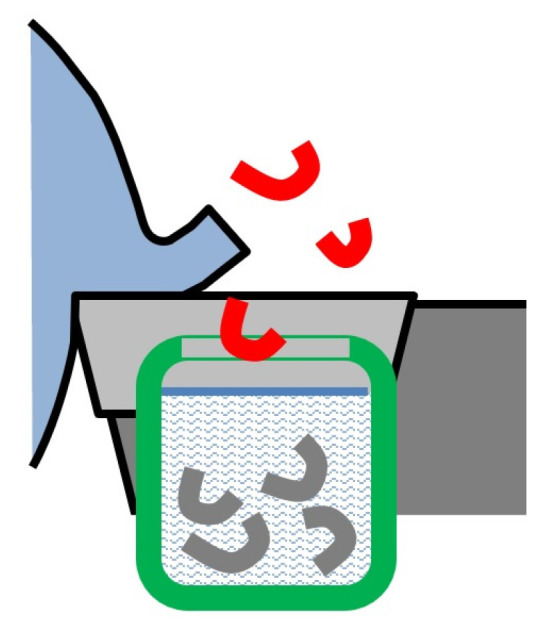
Schematic diagram of the thermal balance (calorimetric) temperature measurement method.

Attempts at the identification of cutting temperatures were some of the first issues addressed by researchers of machining processes. One of the first methods was the **thermal balance (calorimetric) method**, used for the first time in 1798 (Figure 2) [27]. In this method, chips formed directly in the cutting zone are collected in a calorimetric vessel filled with water. Subsequently, following the energy conservation law, and on the basis of the thermal balance, the temperature difference is measured for the vessel with water and for the vessel with water and chips. The result allows the calculation of the amount of thermal energy in the chips, and in turn, of their mean temperature. Some attempts were also made to submerge the entire cutting zone (the tool, the workpiece, and the chips) in the calorimeter. As the number of elements involved in the thermal balance is increased, their impact on the measurement accuracy is negative rather than positive. However, they allow the identification of heat distribution, e.g., in the workpiece, tool. or its edges. This method is simple to use and does not interfere with the cutting process. It is suitable for measuring temperatures for any workpiece and tool materials. Nevertheless, it is mostly of historical value today, as it has low accuracy and allows only approximate estimations of mean chip temperatures.

**Figure 3:**
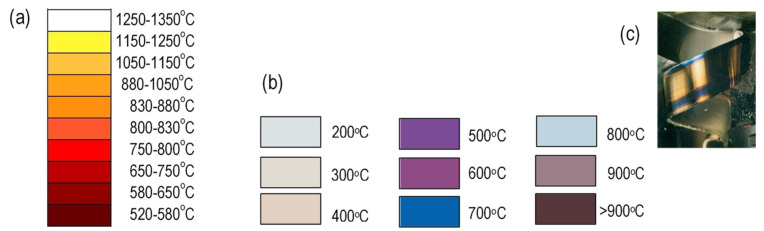
Temperature colors for carbon steel: (**a**) incandescent color, (**b**) chip temper color after cooling, depending on the chip-formation temperature [16], (**c**) view of the bottom part of the chip during its formation (according to Sandvik).

**Chip temperature** can be identified by investigating **chip color** during cutting or after cooling. The more intensive the color change of the chip removed from the cutting zone, from brown–red (520–580 °C) through bright red (800–830 °C) to dazzling white (1250–1350 °C), the higher its temperature value, as shown in Figure 3a [28].

On the other hand, the chip after cooling can have different temper colors depend-ing on the temperature in which it was formed. Chip colors after cooling vary depending on the material type, and in the type of steel (Figure 3b)—even on its grade. Therefore, in order for the temperature measurement to be more accurate, a color range appropriate for a particular material should be used. The reliability of this method is limited [29].

**Figure 4:**
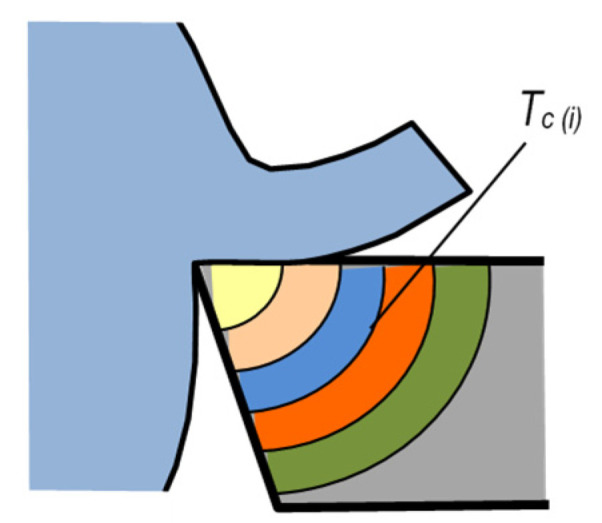
Schematic diagram of temperature measurements with the thermometric ink method.

The next discussed temperature measurement method is the **thermometric paint method** (Figure 4). It is based on the fact that thermometric ink changes its color at a certain temperature. By covering the tool with numerous layers of such inks, it is possible to define temperature distributions on the edge or on the workpiece, or in the slot of the cut workpiece. The disadvantage of the method is that the temperature cannot be measured in the most interesting location, i.e., in the tool edge–workpiece interface zone, as the ink is abrasively removed from its surface. In many cases, the color changes depending not only on the temperature value but also on the duration of exposure to the temperature. The reaction times to temperature also vary. They may be from several seconds to several hours. This fact limits the applicability of the method in dynamic tests. Thermometric inks are applied with brushes, special sticks, or by spraying. The method allows temperature measurements within the range of 40–1350 °C [30].

It is simple and cheap, does not interfere with the cutting process, can be applied to any workpiece and tool materials, and allows isothermal line identification. It was replaced by more convenient, albeit not always more accurate, thermal vision methods.

**Figure 5:**
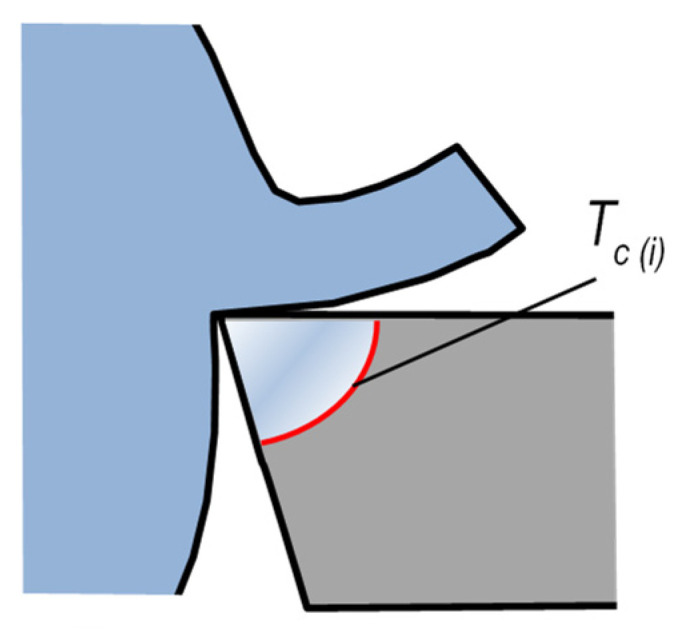
Temperature measured with the boundary melting temperatures method.

A very similar method employs the **boundary melting** temperatures (Figure 5) and consists in the covering of the tool edge with thin layers of different materials having known melting points [31]. Fusible layers are applied to the measured surfaces with the use of special sticks or by spraying, as well as with Physical Vapor Deposition (PVD) techniques. The applied indicator melts at a certain temperature and changes from matt to lustrous. The temperature measurement range of 50–1400 °C is as wide as in the case of the thermometric paint method. It also has similar advantages and disadvantages. Both the thermometric paint method and the boundary temperature method are relatively accurate, down to several percentage points. However, the temperatures are measured outside the edge–workpiece interface.

The method of **metallographic structure changes** consists in the observation of how the structure of hardened high-speed steel changes depending on temperature [32]. The method shows limited accuracy and can be applied only to measurements of steel edges. Advantageously, it allows temperature measurements inside the edge. Its disadvantages, on the other hand, lie in the fact that structural changes in metals depend not only on temperature but also on the duration of exposure to temperature. It is also destructive for the tool edge.

**Figure 6:**
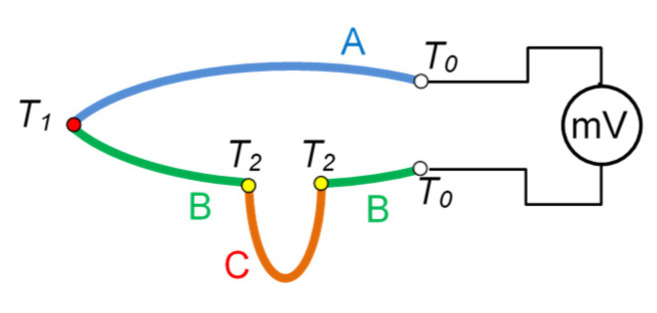
Schematic illustration of the law of intermediate metal.

Methods based on **thermoelectric phenomena** (Seebeck, Peltier, Thomson) employ temperature-dependent electric voltage generated in connected wires made of different electrically conducting materials which form a thermocouple (Figure 6) [33,34]. The greater the temperature difference between the hot junction and the cold junction, the higher the voltage value. Commercially available thermocouples, in the cutting industry referred to as artificial thermocouples, do not require calibration, as they are provided with thermoelectric characteristics, which are advantageously steep and approximately linear [35,36]. The thermoelectric characteristics of different metals are by custom defined in relation to platinum (Pt) [37]. This fact allows thermocouples to be constructed of any material, as the known Pt-related thermoelectric characteristics of each material can be added in order to find the characteristics of the new thermocouple without the need for the calibration process. It is a significant advantage, particularly in cases when one or both thermoelectrodes are natural, being the workpiece and/or the tool. *Natural* and *semi-artificial* thermocouples used in cutting processes must be calibrated, typically in high temperature ranges. Although the Pt-related thermoelectric characteristics of most metals are known, the metals used in cutting processes are typically alloys, and as a result, the calibration process is required [38]. The characteristics of thermocouples from some metals or alloys are non-linear. Moreover, in some temperature ranges, they can change their characteristics from positive to negative, causing potential interpretation problems.

Most cutting temperature measurement methods employ some type of **thermocouples** [39]. Before their basic types are listed, the so-called **law of intermediate metal** should be introduced. It states (Figure 6) that if a section of a wire made of metal C is inserted at any point into an electric circuit comprising metals A and B, and if both ends of this wire have identical temperature, then the thermoelectric phenomena due to the additional thermocouples (BC and CB) will cancel each other and the indications of the thermocouple will not be disturbed. This is an advantageous phenomenon and is employed in a number of temperature measurement methods, particularly in the cutting of metal objects.

**Figure 7:**
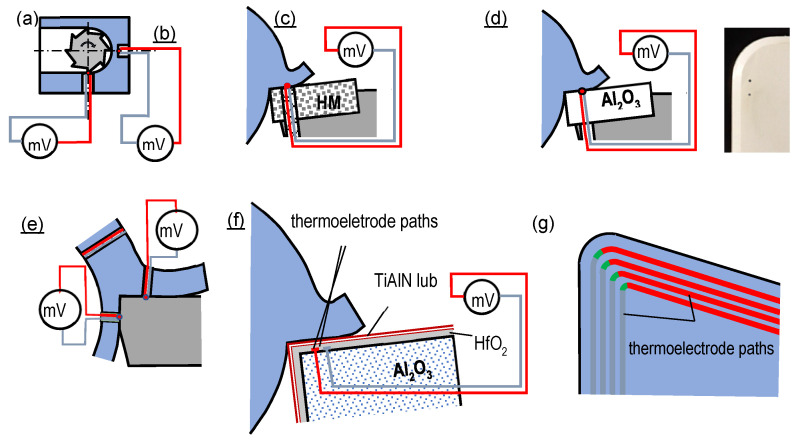
Cutting temperature measurement methods based on the so-called artificial thermo-couple: (**a**,**b**) positioned in the workpiece, (**c**) positioned in the edge of an electrically conductive tool, (**d**) positioned in the edge of an electrically non-conductive tool, (**e**) embedded (single wire), (**f**,**g**) deposited on the rake face of a ceramic edge.

In thermocouple-based methods, the materials of both the tool edge and the workpiece are of significance. When one or both of the materials are electrically conductive, they can be used as one or two natural thermoelectrodes. On the other hand, when the material is dielectric, e.g., ceramic, it can serve as a perfect insulation for the thermoelectrode, even in high temperatures. It can also serve as a base in physical or chemical vapor deposition (PVD, CVD) of thermoelectrodes arranged in the form of thin layers; see Figure 7f,g [40].

The **artificial thermocouple method** (Figure 7) employs a thermocouple of known thermoelectric characteristics, optimally linear, with great temperature range and steep gradient, installed in the temperature measurement location [41].

The advantages of the method include no need for the calibration of the measurement system during the machining process of any pair of the edge and workpiece materials, including dielectric materials, as well as insensitivity to anti-wear coatings, processing fluids, chips, etc., [42,43].

In the method, the greatest problem is to position the hot junction of the thermo-couple as close as possible to the cutting zone. For this purpose, the tool edge or the workpiece is provided with openings or grooves accommodating the hot junction (Figure 7a,b,e). The difficulty lies in ensuring a good adhesion between the hot junction and the bottom of the aperture, as it influences, together with the volume of the junction, the warming time and occasionally also the measurement error. This disturbance is minimized by using pastes or glues with high thermal conductivity. Another problem may result from the need to insulate the thermoelectrodes from each other and from the tool edge and to locate such a thermocouple in a relatively long aperture with a limited diameter or in a narrow groove. Steep temperature gradients in the edges and low thermal conductivity coefficients seem to be the most problematic, particularly in the case of ceramic tools. Also, the aperture in the edge, located in the area of very steep thermal gradients, may significantly disturb the heat distribution, as it affects the temperature distribution in the tool [44]. The above inconveniences may be partially remedied by placing the thermocouple on the depth-of-cut line (Figure 7a,b). When the edge reaches the junction of the thermocouple, the value of the temperature in the particular location of the cutting zone can be measured with a satisfactory accuracy. However, typically, the junction is destroyed in the process. Such an approach also eliminates problems with voltage signal acquisition from the tool or workpiece rotating typically at high speeds.

Another type of the artificial thermocouple method is shown in Figure 7c. In this method, the thermocouple wires can be insulated and inserted into the edge. The chip connects the wires, forming the hot junction [45]. In this method, the constraint results from the requirement to insert the thermocouple wires while ensuring their stability and avoiding the chipping of the tool edge in the vicinity of the wire opening on the rake surface. This procedure is problematic due to difficult conditions related to high unit pressures on the edge and high temperatures and high chip speeds on the rake surface. In another type of this method, the thermoelectrodes are installed directly in the ceramic edge during the sintering process (Figure 7d) [46,47,48]. In this solution, problems with wire insulation are avoided and, moreover, the wires are firmly inserted in the edge, as ceramic material contracts substantially in sintering. An option of regrinding the tool edge allows point measurements of temperatures at various distances from the cutting edge. With this solution, it is possible to define temperature distributions, including maximum temperatures, on the cutting-edge surfaces [49].

Although the method is very interesting, its implementation is problematic, as it requires access to the technology of sintering ceramic tools and allowance for a significant increase in the tungsten grain size during the sintering process (if one of the thermoelectrodes is made of this material), which entails very high brittleness of the wire protruding from the tool edge. This problem can be addressed by polishing the bottom part of the thermoelectrode plate and fastening an additional wire to the polished surface with a special electrically conductive glue. The method allows the identification of maximum temperatures on the surfaces of ceramic edges even in extremely adverse cutting parameters.

The methods which employ the intermediate metal law to form the hot junction and which are presented in Figure 7c–e are limited to some extent by the fact that the workpiece material must be electrically conductive, and the measurement point must be located on the tool–workpiece interface [50].

Positioning thermocouples inside tools is typically complicated and becomes even more problematic during measurements, when the tool rotates. As the rotational speed can be very high, it is difficult to acquire an uninterrupted signal from the tool. Another disadvantage of the method also results from its sensitivity to the dynamics of the tool/workpiece warming process, which depends on the heat conductivity, the process duration, as well as on the distance between the measurement point and the heat source [51].

**Figure 8:**
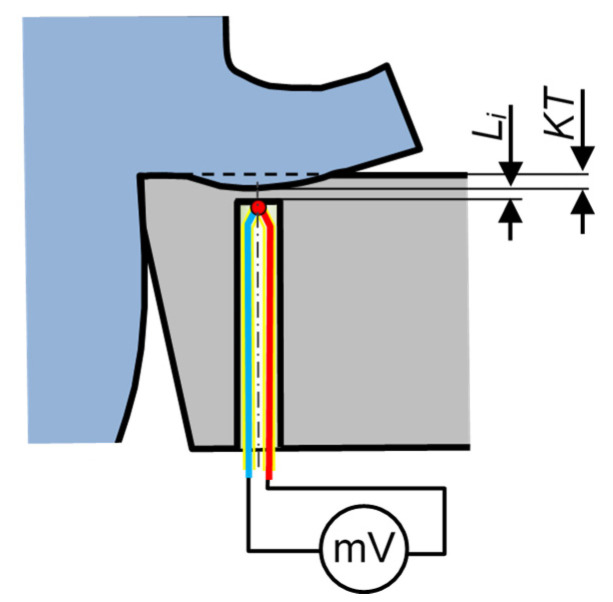
Schematic illustration of disturbances in cutting temperature measurements due to wear on both the *KT* tool and the crater, and the related change of distance *L_i_* from the measurement point to the investigated surface.

The increasing measurement uncertainty is also due to the changing distance *L_i_* between the measurement point and the investigated surface, as indicated by a groove having an increasing crater depth *KT* (Figure 8). For thermal gradients on the order of 2000 °C/mm, a change in the distance between the hot junction and the rake face by only 0.1 mm may modify the temperature reading by as much as 200 °C.

Another type of the artificial thermocouple method is the **embedded** (single-wire) **method**, shown in Figure 7e. It consists in positioning a thin tube with insulated thermoelectrodes in the workpiece. During the cutting process, the thermoelectrodes are cut with the tool edge, and the electrically conductive tool material connects them, creating a hot junction. As a result, two thermocouples are formed. One is lifted together with the chip and moves on the rake surface, and the other is embedded in the workpiece and moves on the flank surface. Theoretically, the method allows the measurement of temperature distribution along the flute on the rake face and also on the flank face [52]. Although the method is very interesting in theory, its implementation is difficult in practice for the following reasons:

- Significant problems transmitting the signal from the rotating workpiece and even more problems transmitting the signal from the thermocouple in the chip, particularly in high-speed cutting conditions;
- No possibility to perform measurements on ceramic tool edges or on edges provided with electrically non-conductive anti-wear coatings;
- Oscilloscopes with short reaction times are needed;
- No practical possibility to perform measurements if the cutting time is longer than several seconds, as it does not allow the cutting zone and particularly the tool edge to stabilize thermically;
- Signal disturbances due to friction between the thermoelectrodes and the edge surfaces, causing significant noise and hindering the insulation of the proper signal value;
- Disturbances of weak thermoelectric signals due to electric current being induced in the thermocouple wires from electromagnetic waves; the problem is observed in a number of measurement methods, as many such waves exist around the measurement stand, including waves from strong fields generated by electric motors in machining and other production equipment;
- Significant measurement problems in non-orthogonal cutting and machining processes other than turning.

An interesting type of the artificial thermocouple method, albeit difficult in practical implementation, is shown in Figure 7f [16]. Thin-film thermoelectrodes are applied to the surface of a ceramic plate with PVD technology. The thermoelectrode paths are protected with an HfO_2_ insulating layer, and all of the layers are protected from abrasion with an anti-wear coating. In this solution, numerous thermocouple paths can be applied to the rake face in order to identify the temperature distribution on the tool edge (Figure 7g). Both of the above solutions have a significant disadvantage in that they are very sensitive to wear on the tool edge, in the form of a groove on the rake face, and can quickly destroy the junction of the thermocouple. Moreover, the solutions can be applied only in the case of turning with the use of ceramic tool edges. The advantages are a small measurement point and the possibility to identify temperature distributions on the edge and also perform measurements when machining dielectric, including composite, materials. Once calibrated, the thermocouple allows measurements to be performed during the cutting process of any material. The method does not disrupt the cutting process.

**Figure 9:**
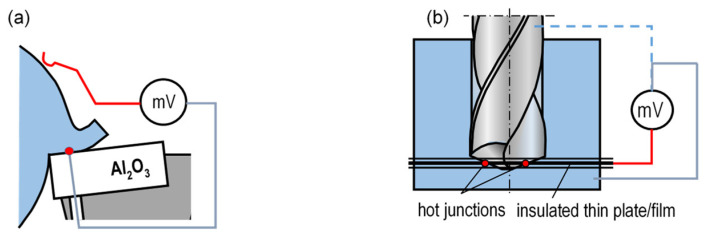
Schematic diagram of cutting temperature measurement with the use of semi-artificial method as related to: (**a**) turning, (**b**) drilling.

The next direction in the development of thermocouple-based methods was to replace one of the artificial thermoelectrodes with a natural thermoelectrode, i.e., with the tool or the workpiece material. The types of this method, referred to as **semi-artificial** methods, are shown in Figure 9 [53].

**Figure 10:**
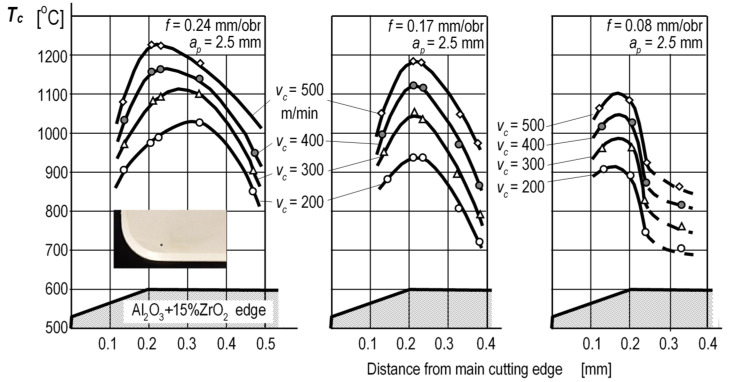
Temperature distribution on the rake surfaces of ceramic edges during the process of cutting medium-carbon steel [7]. Distance from main cutting edge [mm].

In the method shown in Figure 9a, the natural thermoelectrode is effected by the workpiece and the artificial thermocouple—by a thin wire made of, e.g., tungsten or platinum. Interestingly, the thermocouple wire, 0.05 mm in diameter, results in a measurement point having a surface of ≈0.002 mm^2^. This fact allows point measurements of temperature on the cutting-edge surfaces. The method is similar to the method presented in Figure 7d, and thus it has similar advantages and disadvantages. Figure 10 shows representative temperature distributions on the rake face obtained with this method.

In the semi-artificial method used in drilling (Figure 9b), the artificial thermoelectrode is an insulated thin plate/film arranged between two parts of the workpiece material [54]. The natural thermoelectrodes may comprise the workpiece or the tool material. The hot junction of the thermocouple comprises points on the interface between the cutting edge of the drill and the plate or between the workpiece material and the plate. The version in which the thermocouple comprises the drill and the plate is more difficult to implement, as the signal must be acquired from the rotating tool, and is thus not used. The advantage of the method lies in the possibility to define temperature distributions along the cutting edges. The measurement is performed correctly on the condition that the plate is sufficiently thin and insulated and does not interfere extensively with the heat distribution around the hot junction. The measurement process is relatively simple, although it requires calibrating the measurement thermocouple upon every change of the workpiece material.

**Figure 11:**
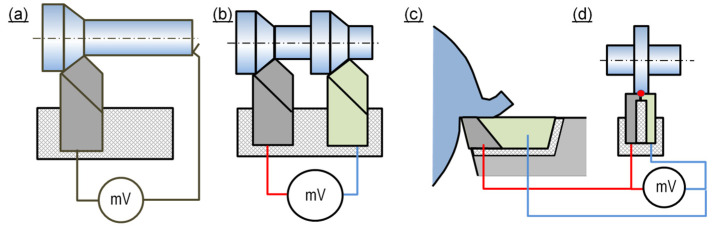
Schematic diagrams of cutting temperature measurement with the use of natural thermocouple method: (**a**) single-edge, (**b**) double-edge, (**c**,**d**) split edge in two varieties.

The **natural thermocouple method** is based on the fact that the workpiece and the tool are made of electrically conductive materials and can be thus used as thermoelectrodes in the thermocouple (Figure 11). In its **single-edge** type (Figure 11a), the method is advantageously very simple and not time- or labor-intensive. Its disadvantages include the inconvenience of calibrating non-standard thermocouples and of insulating the workpiece and the tool from the lathe, and also potential measurement disturbances from short circuits produced by chips [55]. The method is used to measure mean temperature values from the entire edge–workpiece interface. Measurements following this method show significant limitations if the edge or any layer of the anti-wear coating is a dielectric. Another difficulty results from the need to acquire the voltage signal from the rotating tool. The **double-edge** solution (Figure 11b) is similar to the single-edge type, with the difference resulting from the fact that the second thermoelectrode of the thermocouple comprises the second, twin tool, which is manufactured of different material. In accordance with the intermediate metal law, the workpiece does not significantly interrupt the measurement. This is true, however, only if the operating conditions are possibly similar for both edges. The difficulty is that both tools should be made of materials having different thermoelectric characteristics, so that they form a thermocouple of a certain sensitivity. This constraint causes a problem, as the material separation conditions should be similar for the two different edges. Therefore, the method can be applied only in the case of approximate and comparative temperature measurements. The advantage of the double-edge method over the single-edge method is in that the thermoelectric characteristics of such a thermocouple can be identified once for both of the tool materials and the measurements can be performed during the cutting process of various, importantly conductive, materials. A variant of this method, referred to as “split edge” and shown in Figure 11c, allows investigations of temperature distributions on the rake surface, if the two tool edges are connected at a distance different from the cutting edge.

In the solutions shown in Figure 11c,d, no need exists to acquire the signal from the rotating tool or to calibrate the thermocouple upon each change of the workpiece material.

**Figure 12:**
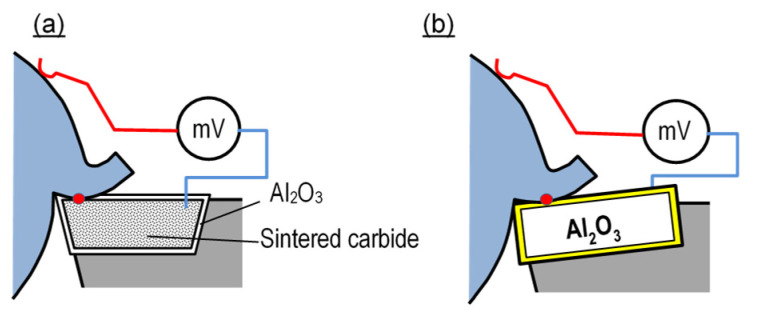
Schematic diagram of cutting temperature measurement method using a single-edge natural thermocouple and based on different properties of edge-protecting coatings: (**a**) dielectric coatings, (**b**) electrically conductive coatings.

The group of natural thermocouple methods includes those solutions which employ different properties of edge protective coatings (Figure 12). If an edge made of, e.g., cemented carbide is covered with a dielectric coating (Al_2_O_3_) (Figure 12a), the natural thermocouple is formed when the coating is compromised in use. The method is advantageous in that it allows measurements of maximum temperatures on the edge, which are typically observed at locations showing the most intensive wear. A problem may occur if the coating is compromised not on the rake face but rather on the flank face, or due to the chipping of the cutting edge. Moreover, note should be made that increasing wear causes the area of the hot junction to increase, changing a point-type measurement into a medium-surface measurement type.

In the case of ceramic edges (Figure 12b), it is possible to use a natural thermocouple only if an electrically conductive layer is deposited on the edge surface. In this method, a reverse tendency is observed: the initial measurement provides mean temperature from the entire tool–workpiece interface, and after the conductive layer is compromised, the measurement area is limited, omitting the worn part of the surface coating, which typically experiences the highest temperature. A significant disadvantage of the two methods is that the cutting conditions apply not to the carbide or ceramic edge, but rather to the coatings deposited on these materials. Another disadvantage results from the need to deposit the coatings and define their thermoelectric characteristics upon every change of the tool or workpiece material. This fact applies in particular to the method of Figure 12b. Yet another difficulty is caused by the fact that the signal is acquired from a rotating tool or workpiece, if any of them rotates.

The methods shown in Figure 9, Figure 11 and Figure 12 are limited in that they cannot be used in measurements during the machining of dielectric materials.

The cold junction reference temperature *T_0_* does not need to be constant, normally at 0 °C or 20 °C. The reason is that the measured cutting temperatures are typically above several hundred degrees Celsius, and therefore, the disturbances of electromotive force (emf) from small temperature variations on the cold junction are negligible. The entire edge, including the cold junction, may significantly warm and thus affect the measurement results only if diamond or boron nitride replaceable inserts are used, as they have very high thermal conductivity. This problem can be solved if the voltage signal is acquired from an insulated insert connected at one point with another insert of identical material. In such case, the cold junction of the added insert will be exposed to limited heat [56].

**Figure 13:**
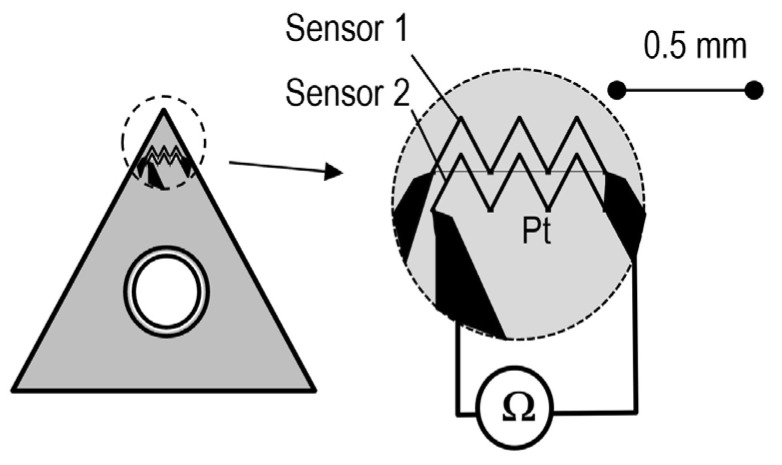
Temperature measurement with the use of the electric resistance method (on the basis of [49]).

**Change of electric resistance** as a function of temperature can be used in resistance thermometers. The sensitivity of the method can be improved by employing temperature-sensitive resistors in which the temperature rise causes a steeper decrease in the resistance [57] of the semiconductor than the increase in the resistance of a regular resistor. However, the measurement range of thermistors on the order of 300 °C is definitely insufficient to render them applicable in measurements of cutting temperatures. The use of a resistance thermometer in a version shown in Figure 13, with platinum resistance wire, allowed the measurement range to be extended significantly above 1000 °C (the melting point of Pt is 1768 °C). A significant disadvantage of the method results from the complicated deposition of a miniaturized Pt resistance layer and in the necessity to effectively protect this layer against damage during the cutting process. Despite considerable miniaturization of the measurement zone, the method does not allow measurements of temperature distributions on the edge, and instead provides only mean values from a defined field. On the other hand, the method advantageously does not require calibration, as the thermometric characteristics of Pt are known [58].

**Infrared radiation (IR) emission** is, on occasion, used in surface temperature measurements performed with pyrometers or thermal vision cameras. This is the method most typically employed in cutting processes, and it has numerous different implementations [59]. Methods based on analyzing surface emissivity are advantageous in that the measurement is remote; it does not disrupt the investigated process, including the heat distribution; and it allows the identification of temperature distributions [60].

A certain problem is that the value of emissivity *ε* for a particular surface depends not only on temperature but also on numerous other factors, such as the length and direction of the radiation wave, the roughness and the color of the surface, the type of material, and the oxidation degree. Typically, the above constraints necessitate the calibration or definition of emissivity characteristics for tested objects to be performed within high temperature ranges. These procedures need to account for/eliminate the possibility of surface oxidation during the calibration process, as there is insufficient time for this oxidation to occur during a dynamic machining operation. A surface oxidized during the calibration process may have a substantially greater emissivity than an unoxidized surface. For example, depending on the condition of its surface, differences in the emissivity for steel may be significant, as demonstrated in Table 1. Without allowing for this fact, a correct temperature identification may be significantly imprecise [61].

Importantly, when measuring the radiation rate of an electromagnetic wave, record is made not only of the wave emitted by the surface of the investigated body, but also of the radiation reflected from the surface, and—in the case of transparent bodies—also the wave passing through the surface. In effect, the detector of the IR camera records the so-called *apparent temperature*, which is uncompensated. The *true temperature* of the surface is due to only molecular movement in atoms and particles. In the case of high surface reflectivity, the reflected wave may have a significantly higher radiation rate than the wave emitted by the surface. Therefore, this type of measurement should be performed at a distance from strong IR sources, such as lighting fixtures, sun, heaters, etc., particularly within the wavelengths received by the matrix of the IR camera. Otherwise, the temperature measurement can be imprecise. In order to find the true temperature of an object, the apparent temperature must be compensated. In this process, particular attention should be paid when the measurement is performed for a surface with very low emissivity (bright, glossy), as its apparent temperature becomes similar to the ambient temperature. A glossy object will seem colder in lower ambient temperature, and hotter in higher ambient temperature, as the camera will record more of the radiation reflected rather than emitted by the object [63].

In general, emissivity-based surface temperature measurements are more difficult in the case of metals than non-metals. The reason is that metals, in particular those with smooth surfaces, have emissivity levels on the order of several hundredths of unity, and non-metals, close to unity (*ε* = 1).

**Figure 14:**
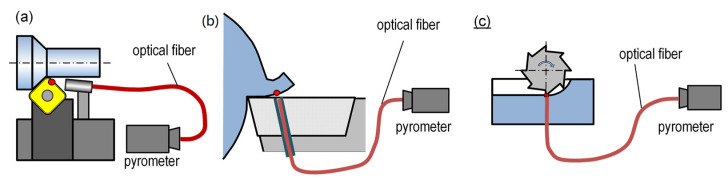
Temperature measurement with a pyrometer positioned (**a**) as close as possible to the cutting zone, (**b**) in the cutting edge, (**c**) in the workpiece.

To some extent, the effect of radiation-induced disturbances of the measurement can be overcome by using a pyrometer and optical fiber, if its end can be positioned as close as possible to the measurement point. Such a solution can be used in methods shown in Figure 14.

**Figure 15:**
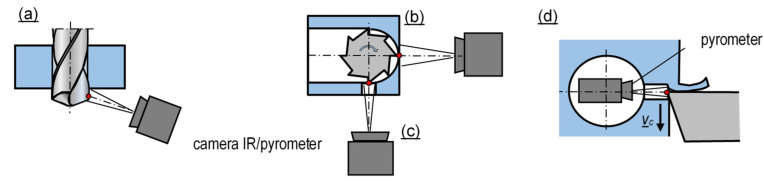
Temperature measurement based on IR emission and exposed edge surface: (**a**) directly after the tool exits the workpiece, (**b**) at the moment of entering the workpiece, (**c**,**d**) through an opening arranged in the workpiece [56].

However, difficult access to the cutting-edge surfaces, whose temperature is typically of the greatest concern, necessitates the implementation of further solutions. Some of them are shown in Figure 15. In the case of rotating tools (drills, end mills) engaged in the workpiece, the temperature can be measured immediately after the tool exits the material (Figure 15a,b), thus minimizing the temperature decrease of the edge. The tool surfaces can be also exposed by means of openings arranged in the workpiece (Figure 15c,d).

**Figure 16:**
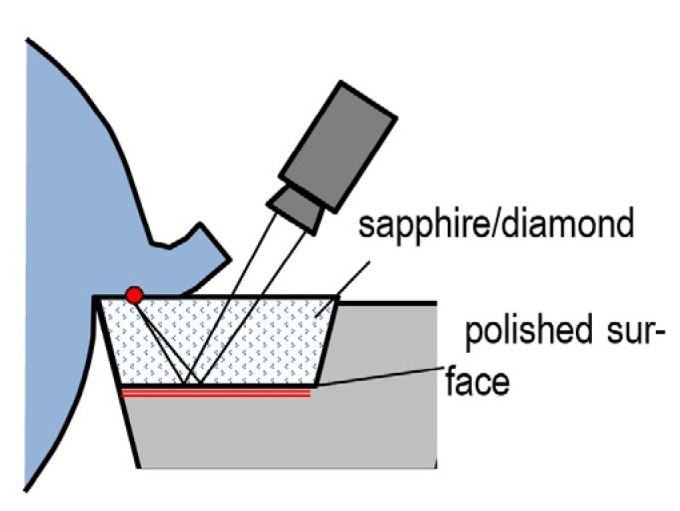
Temperature measurement with the use of a transparent edge.

An original, albeit complicated method employs transparent edges made of sapphire monocrystal or diamond (Figure 16) [64]. In this method, a pyrometer can be used to measure the temperature distribution on the bottom surface of the chip interfacing the rake surface. This is most likely the sole advantage of the method, which is therefore not commonly used. Its main disadvantages include: the need to use very expensive edge material [65], which is difficult to shape; problematic identification of the IR absorption coefficient; disturbances due to chips and processing fluids; and different cutting conditions and heat distribution due to the specific edge material.

In many cases, impressive, colorful thermal vision images of investigated objects do not adequately represent their real measurement quality. The source thermal vision image is actually obtained in shades of gray. These shades are translated into individual colors at the stage of computer processing in order to add clarity to the image.

**Figure 17:**
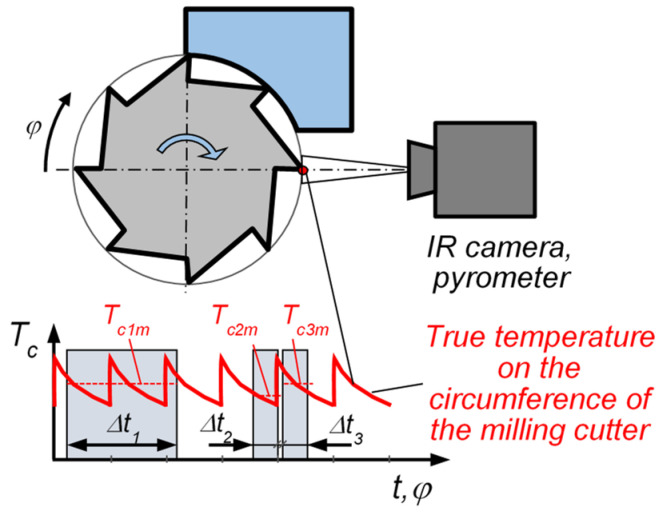
Schematic representation of temperature mismeasurement in the case of rapidly changing temperatures and different exposure times Δ*t_i_*.

Cutting temperature measurements with the use of methods based on emissivity are easier, if this is measured from “stationary” locations, which are understood as such that have limited or no displacement dynamics, e.g., from the edge of the turning tool, and also when the investigated surface moves very fast, but has an identical quasi-constant temperature, as in the case of, e.g., the bottom part of the chip removed at a certain distance from the cutting edge. On the other hand, for example, in the case of a tool rotating with high speeds and showing diverse temperature distributions on the surface, a temperature measurement at a certain point of the tool is very difficult. This difficulty is due to the response time of the measurement system. The thermal vision image of an excessively quickly moving object will be recorded with a blur (out of focus), and the temperature value will apply not to the point but rather to the mean value from a distance traveled by the object during the exposure of the IR camera or the pyrometer [66]. Figure 17 schematically shows such disturbances. The continuous line indicates the actual temperature distribution along the circumference of the rotating tool. The dashed line indicates the mean temperature *T_ci_* from the signal recorded at various time intervals Δ*t_i_*. In this example, the temperature values measured with the use of the thermal vision camera/pyrometer are different (*T_c1m_* ≠ *T_c2m_* ≠ *T_c3m_*) and depend on the moment and duration of exposure, as well as on the data acquisition method from the camera matrix. In rapidly changing temperatures, the exposure time for individual frames of the IR camera is of great significance. Without allowance for this fact, temperature measurements for a particular point may be very imprecise. The majority of simple thermal vision cameras let the images be recorded at a frequency from several to tens of Hz, and this fact significantly limits their usability in dynamic temperature measurements. Some thermal vision cameras (e.g., manufactured by InfraTec, Dresden, Germany) allow recordings at a frequency of up to 105 kHz. Some IR cameras allow the recording frequency to be increased by narrowing their observation field on the measurement matrix, thus limiting the number of acquired pixels on the thermosensitive matrix. Due to very short exposition times in such cameras, signals from the measurement matrices have very low values. In order for the noise value not to interfere significantly with the measurement, such a matrix needs cooling, typically with liquid nitrogen [67].

Interestingly, IR cameras are optionally provided with an additional adjustable release mechanism controlling the exposure (e.g., with an accuracy of 10 ns), which can be used to synchronize the image recording frequency with the frequency at which the investigated fragment of the tool edge is exposed. This option allows a significant reduction of the measurement uncertainty in the case of rapidly and cyclically changing temperature.

Measurements of rapidly changing surface temperature distributions are signifi-cantly more labor- and cost-intensive due to more expensive apparatus; in addition, they are not always technically feasible [68].

In measurements of particularly rapid temperature variations, a difference needs to be made between two notions: *imaging frequency* and *exposure time* for individual frames of the IR camera. The second value is of greater importance, if accurate measurements of local and highly dynamic temperatures are required. For example, if the cutting process is performed with speed *v_c_* = 500 m/min, and the exposure time of the camera is only 0.001 s, the object will be displaced by 8.3 mm during the measurement. The camera will thus measure the temperature not at one point, but will most probably provide an approximate mean value from the length of the line [69]. If the temperature is measured on the circumference of a milling cutter, the exposure time will be sufficient to measure mean temperature on several edges and chip flutes, and therefore the result may be far from expected. However, if a very good quality camera is used, with a record frequency of 28 kHz and the corresponding exposure time of at least 0.03 ms, the object with speed *v_c_* = 500 m/min will be displaced only by approximately 0.3 mm.

In many cases, if temperature measurements are performed during a high-speed cutting or for hard materials, and if an IR camera is aimed at a zone in which the chip is white or light yellow (i.e., at a temperature of 1200–1300 °C), the temperature indicated by the IR camera can be lower by as much as a half [70]. This phenomenon may be due to the above-discussed reason, or may be attributed to the highly reflective smooth surfaces of the chips, which reflect a considerably lower ambient temperature. The limited area of the high-temperature surface may also be the reason, as it insufficiently covers the pixels in the IR matrix [71].

Problems with identifying rapidly changing temperatures, particularly on surfaces moving at high speeds, are the reason why the vast majority of publications which address this issue focus on orthogonal cutting and fixed edge methods [72,73].

Two-color pyrometers eliminate the need for calibration, and more specifically, for the identification of emissivity characteristics for the analyzed surface. This fact is a significant advantage.

Pyrometers are considered tools for point measurements of temperature. Even if the mathematical definition of a point is expanded and if it is assumed to include a very limited surface area, still, the notion of *point measurement* can be significantly overinterpreted in relation to a pyrometer, or an IR camera [21].

**Figure 18:**
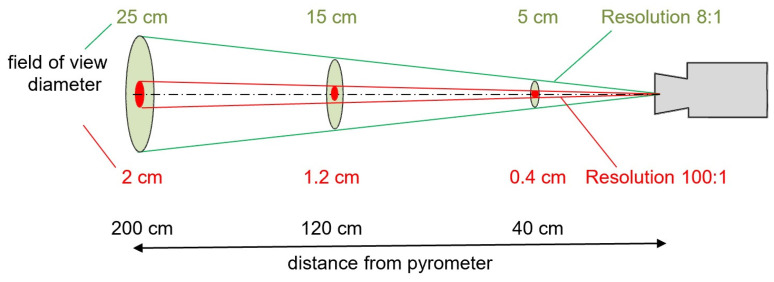
Pyrometer field of view sizes depending on the optical resolution and the distance from the pyrometer.

Pyrometers have the so-called *optical resolution*, which is defined as the ratio of the distance between the detector and the measurement object to the diameter of the circular field of view, as illustrated in Figure 18. For example, in the case of an 8:1 optical resolution, the field of view diameter for a “point” at a 2 m distance is 200:8 = 25 cm, and such an area is not normally referred to as a point. Typical pyrometers have optical resolutions from 3:1 to 100:1. Figure 18 shows representative pyrometer field of view sizes depending on the optical resolution and the distance from the pyrometer [74,75].

**Figure 19:**
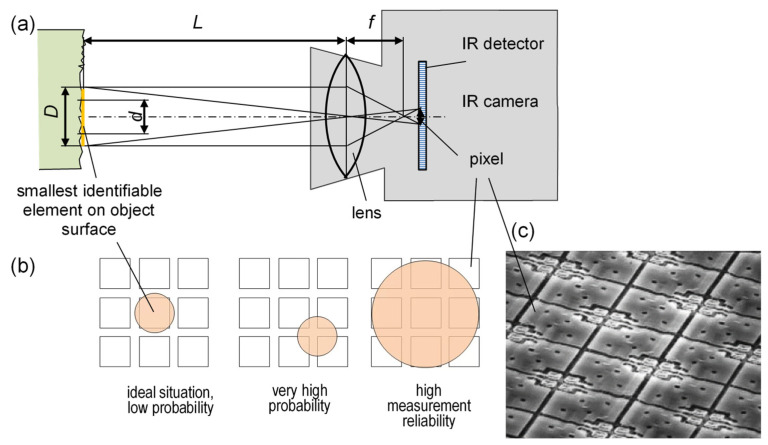
Schematic illustration of defining the smallest fragment of the surface d determined by the size of the detector pixel, the focal length *f*, the IR camera matrix, and the distance *L* between the object and the camera: (**a**) location of the element of the investigated surface relative to the pixels of the IR detector, (**b**) fragment of a thermosensitive bolometer matrix (**c**).

The case is similar for IR cameras. Their thermosensitive matrix pixels (typically 7–50 μm in size) may reach significant diameter D on the measured surface, depending on the focal length f and the distance L of the object from the camera. Conversely, an image of the smallest segment d of the observed field does not have to be represented centrally on the matrix pixel (Figure 19b) [76].

Insufficient field size *d* of the observed surface relative to the diameter D which allows a reliable temperature measurement (Figure 19) may significantly affect measurement reliability. This fact applies in particular to methods presented in Figure 15c,d. Therefore, in order to measure a temperature from a small surface, the camera should be moved as close as possible to this surface and provided with a high-resolution lens, particularly with high-resolution IR matrices. Various designs of IR cameras may employ different methods of assigning a temperature value to a matrix pixel. Typically, the value is not measured from one pixel, but rather as a mean from e.g., nine surrounding pixels [77,78].

**Figure 20:**
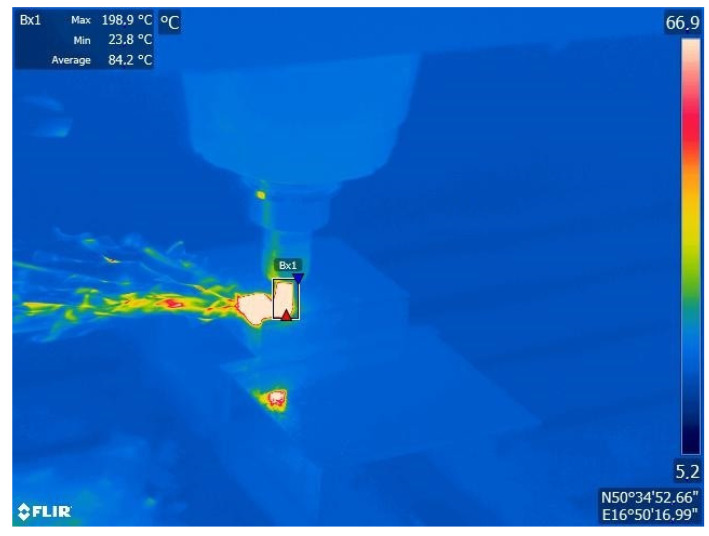
IR camera screen during temperature measurements of cemented carbonite mill edge in the process of cutting hardened steel.

Modern IR cameras have low-resolution matrices 320 × 256, 480 × 380, 640 × 512, or 1920 × 1536. These values are substantially smaller than in photographic cameras. Measurements with IR cameras are most reliable when the surface fills the matrix/screen to the greatest degree. In the case of temperature measurements, the procedure first requires the identification of the area with the highest temperatures (Figure 20) and, in the next step, moving the camera closer to the object or applying greater focal lengths in order to limit the observation field, so that the investigated image fragment occupies the largest possible fragment of the thermosensitive matrix [79].

Importantly, the results may be significantly disturbed by the transparent screen used to shield the measuring apparatus from the hot chips or heat during the high-temperature calibration, as most of technical glass types have emissivity values *ε* = 0.9–0.95, which cause them to be practically non-transparent to IR cameras [80,81].

However, it is possible to use a shield from, e.g., germanium or another material transparent to the IR wavelengths recorded by the camera matrix. Typically, such shields are made of a material identical to that in the lens and can also be installed in the furnace door for use during the process of surface emissivity measurements in high temperatures.

Reflections from “cold light sources” such as LEDs or neon lights do not produce significant disturbances, as most of their energy is transformed into the visible light rather than infrared radiation.

Measurements in which the object is insufficiently small relative to the size of the IR camera may be imprecise, as is the case, e.g., in the temperature measurements of the tool edge during the cutting of hardened steel shown in Figure 20. The results indicate the highest recorded temperature at 256.1 °C, but the actual value was several times higher.

Most errors in IR-based temperature measurements result from misestimations of emissivity and from its low value [82,83].

**Figure 21:**
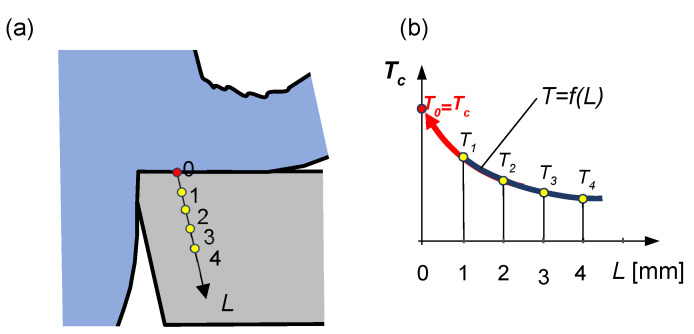
Schematic representation of temperature measurements based on the extrapolation method: (**a**) distribution of measurement points inwards the edge material, (**b**) temperature extrapolation outside the region of the experiment.

**Extrapolation methods** are part of experimental-calculation methods. Most such methods consist in measuring temperatures at different distances from the investigated point [84]. In the next step, a function is defined to show the relationship between the temperature change and the distance, and its curve is extrapolated to the investigated location (Figure 21) [26].

**Analytical methods** may employ mathematical and physical models of the cutting process. *Mathematical models* are generated on the basis of experimental tests, describing the influence of cutting conditions or parameters typically with an exponential or a polynomial function [85]. This function can be subsequently used to calculate the cutting temperature value. The model is valid only for the condition ranges in which the prior experimental tests were performed [86].

The defining of cutting temperatures based on *physical models* of the cutting process is a more complex issue. It employs, e.g., the finite difference method (FDM) or, more commonly, the finite element method (FEM), which require substantial computing power as well as the knowledge of numerous boundary conditions [87]. The latter are, on some occasions, very difficult to identify precisely [88].

Regardless of the analysis method, both the analytical modeling and the FDM and FEM require information on the specific heat, thermal conductivity, diffusion coefficient, and material density as a function of temperature. One of the fundamental problems is to identify correct thermal and physical properties in a relatively wide temperature range. Frequently, additional experiments are required for that purpose [89].

Edge-protecting anti-wear coatings are one of many problems when identifying cutting temperature distributions with analytical and physical methods [90]. Often consisting of many layers, such coatings have physical and chemical properties different than those of the tool material. Such properties as emissivity, thermal and electric conductivity, electromotive forces, friction coefficients, abrasibility, etc. change the material cutting mechanisms, the amount of generated heat, and its distribution proportions [91]. Moreover, anti-wear coatings are subjected to relatively quick local damage. In some situations, as little time as several seconds to under one minute suffices for one or more such coating layers to become compromised. In the case of multilayer coatings, it is very difficult to identify the conditions and interactions between the phenomena occurring in the cutting zone. The problem may be solved by introducing a so-called alternative coating [92,93].

Another problem in analytical methods results from insufficient knowledge re-garding the conditions of heat removal from the cutting zone, when different processing fluid application methods are used (immersion cooling, high-pressure jet cooling, coolant application at different angles and to different areas of the cutting zone, minimum quantity lubrication (MQL), etc.) [94,95].

Calculation methods based on physical models are undoubtedly advantageous in that they allow investigations and analyses of heat and temperature distributions in the cutting zone, in different machining conditions and without effort needed for experimental tests. However, they require a complicated mathematical apparatus and this must be supported by extensive knowledge and computing power [96,97,98,99].

**Figure 22:**
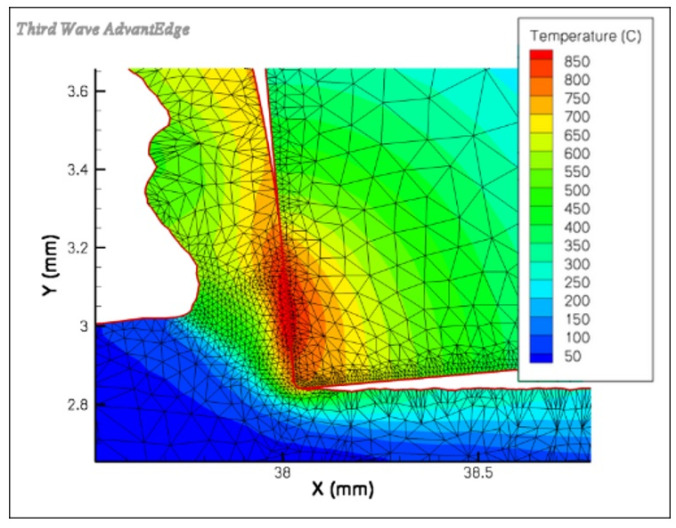
Temperature distribution map during the simulation, in the AdvantEdge software package, of machining steel C45 with a cemented carbonite TiAlN-coated edge for *v_c_* = 106.9 m/min, *f* = 0.1 mm/rev [100].

Figure 22 shows a temperature map for the cutting zone during the simulation of machining steel C45 with a cemented carbonite TiAlN-coated edge, as performed with the AdvantEdge software.

## 3. Discussion

This article has defined terms related to various types of cutting temperatures. Their measurement methods have been extensively reviewed. The technical and application potential of these methods, as well as their advantages and disadvantages, have been also discussed. The presented observations may allow a more informed selection of the measurement method most suitable for the particular situation and objectives.

The large number of cutting temperature measurement methods is the result of the very nature of the machining process: limited cutting zone area, limited accessibility to this zone, high dynamics of the process, and a number of physical and chemical phenomena influencing temperature measurements. Each of the presented measurement methods has its conditions and sometimes very narrow scope of application. Therefore, the selection of the optimal method requires consideration for various—and, in some cases, contradictory—criteria.

Each measurement method has its application area as well as mutually exclusive limitations. For example, although the thermometric paint method, the boundary melting temperatures method, or the metallographic structure changes method are simple and inexpensive, they are not suitable in dynamic tests.

The thermocouple-based methods are used in numerous versions which entail numerous different properties. Some of these methods allow measurements of mean temperature values from the entire edge–workpiece interface, while others have an advantageously small measurement point, enabling precise measurements of temperature distributions, and even maximum temperatures, on the cutting surfaces of the tool. Natural or semi-artificial thermocouples typically require calibration upon every application, while artificial thermocouples do not have this disadvantage. The majority of difficulties encountered during thermocouple-based measurements result from the problematic positioning of temperature sensors as close as possible to the locations on the edge where the highest temperatures are observed. Openings or slots accommodating the thermocouples may disturb the heat distribution and accelerate the tool wear.

The most important contribution of this work is that all of the cutting temperature measurement methods are discussed from the perspective of their usefulness in different applications. Particular attention has been given to the potential of the currently most common IR-based methods. The popularity of thermal vision methods results both from the uncomplicated measurement procedure, which typically does not interfere with the cutting process, including with the generation and distribution of heat in the cutting zone, and from the possibility of performing measurements for various tool and workpiece materials. However, many publications do not fully acknowledge the limitations of the methods based on thermal vision. In many cases, impressive thermal vision images do not allow the correct identification of temperature values. These may significantly differ from the actual values. A significant limitation of thermal vision cameras results from the very difficult access to the edge–workpiece interface. Also, it is problematic to identify surface emissivity in proper conditions regarding roughness, color, reflectivity, oxidation degree, etc. Reliable IR-based temperature measurements for elements of very limited surface area and in highly dynamic processes require high competences, as well as expensive cameras and apparatus.

Knowing certain technical limitations and using equipment with certain measurement parameters, it is possible to modify the objective of the measurement, e.g., instead of identifying the maximum temperature for a particular location on the object, a mean value can be found for a particular area or distance. Also, an optimal method and apparatus may be adjusted to the particular objective. The above analyses may allow the definition of further research directions into the applicability of new measurement methods or new functionalities enabled by improvements to the currently available methods.

This article also points to the possibility of measuring cutting temperatures with the use of analytical methods, based on mathematical and physical models. In some situations, these require additional experimental tests, particularly in relation to various thermometric properties of the tool or the workpiece materials. The FEM/FDM methods implemented for this purpose require expert knowledge and computing power. However, they are very universal and allow the modeling and analysis of temperatures in many variants of machining processes.

## Figures and Tables

**Table 1:** Emissivity *ε* values for steel depending on the condition of its surface [62].

Polished steel	0.07
Freshly rolled steel	0.24
Unpolished steel	0.96
Highly oxidized steel	0.88
Corroded steel	0.69

## Data Availability

The data presented in this study can be obtained by contacting the corresponding author upon request.
